# A Winter in the Upper Engadine, 1885

**Published:** 1913-12-20

**Authors:** 


### A WINTER IN THE UPPER ENGADINE, 1885.

### By One of the First Consumptives to Visit the High Alps.

[The following MS. has come unexpectedly into our hands after being buried for nearly a generation in the privacy of a writing-desk. It has historical interest from being an authentic account, by one of the first English consumptives treated on open-air and sunshine principles, of a Swiss winter, and though it "dates," in the sense that much of its information is now common property, it dates in an amusing way. For instance the detailed description of tobogganing shows what a foreign unbelievable sport this was to English people thirty years ago. What would the writer have thought of the modern four-seated bob-sleigh, and the artificially prepared run, on which hundreds of pounds are spent? For this reason we print the description unedited and uncurtailed as giving a "patient's point of view" worth resurrecting.—Ed. The Hospital.]

It is possible that some account of my personal experiences of a winter in the Upper Engadine, where I was ordered by my medical adviser, may not be without interest, at least to those who have only visited Switzerland in the summer.

### The Patient's Expectations.

At the first blush the mere idea of spending the winter in a region of ice and snow is anything but an attractive one; and, indeed, I started on my journey with feelings the reverse of pleasant, which, however, were to a great extent dispelled when I found myself at the end of the third day's journey in a large and comfortably warmed hotel at St. Moritz, 6,000 feet above the sea-level. Things were not, after all, as cheerless as I had pictured them, for the snow had not as yet put in an appearance, and the lingering autumn tints were still to be seen in the woods clothing the lower slopes of the mountains. The hotel stands as it were at the side of a valley and is completely shut in by the surrounding Alps, which are mirrored by the greenish waters of the St. Moritz Lake lying far below.

My first impression of strangeness speedily wore off, for I soon found that there was much pleasant English society at the hotel, mostly consisting, however, of those who, like myself, had come to seek renewed health. Naturally a friendly fellow-feeling soon sprang up amongst us, and we felt before long as if we had known each other for years.

### The Woods of St. Moritz.

The first few weeks of my out-of-door life were spent in rambling, in company with some of my newly made acquaintances, through the woods with which St. Moritz is fringed in nearly every direction; and they were indeed scenes of unsurpassed loveliness. Many of the deciduous trees had changed to a brilliant golden hue, and these were set off to the utmost advantage by the more sober tints of the ever-green pines, of which the woods mostly consist. There are but few birds in these woods, and the solemn silence pervading them was but rarely broken by the roar of some mountain torrent or the monotonous tapping of the large woodpecker, who was often to be seen clinging to the trunks of the trees in busy search for his insect food.

At length, however, the frost began to set in. An old habitué of the place stated that in a few days the ice on the Statzer-See—a lake about two miles off—would bear. Accordingly our spirits rose in expectation, for it is a very unusual thing in the Engadine to have lake-skating. The snow generally comes concurrently with the frost, thus destroying the smoothness of the ice. But ultimately our hopes were realised and the ice reported safe.

### The Beginning of Skating.

The road to this lake wound down the steepest of steep hills and was of the roughest description. Many of the party, myself amongst the number, wishing to spare their limbs, chartered what the hotelkeeper was pleased to call carriages—but *what* carriages! There seemed to be no springs, and, what with the immense unevenness of the way, the jolting was so great that several of us were almost pitched out, and involuntarily embraced each other in our frantic efforts to keep our seats. One shy young man, who sat by the driver on the box, fell back in the most tragically comic manner imaginable into the arms of a stout good-humoured lady, who laughed so much at his discomfiture that the tears ran down her fat cheeks. But our fears, which doubtless would have been shared by a sympathising onlooker, were set at rest for the time being when our "carriage" got on to a smooth level piece of grass skirting the end of the St. Moritz Lake, across which the most perfect view of distant snow mountains was to be had. Our road then bore away to the left through scattered brush-wood. After a good deal more jolting, and merrymaking in consequence, suddenly the view of the Statzer-See burst upon us. We could but pause to admire the extreme beauty of the scene. The lake lies embedded in woods, and at its back towers the magnificent Piz Languard, whose summit was whitened by the winter snows. The merest powdering of frost rested on the neighbouring foliage, not sufficient, however, to conceal its richness and variety of colouring, and the shadows cast by the woods across the lake threw into bold relief the dazzling whiteness of the snows resting on the surrounding peaks, which one by one caught the slanting rays of the morning sun.

### A Comedy on the Ice.

We alighted from our unpleasnt conveyances congratulating ourselves that we had made the journey without any broken bones. There was little time lost in getting to work. Even our stout friend before mentioned was most anxious to distinguish herself on the ice; and, indeed, but few ladies were content to pose as inactive spectators. Some few of us, however, who were disposed to take matters somewhat easily set ourselves to explore the nooks and corners of the lake and were well rewarded for so doing. The ice was as clear as crystal and of a delicate green tint. So transparent was it that we could distinctly see the variously tinted aquatic plants which grow luxuriantly many feet below the surface of the water, and sometimes shoals of small trout, which, however, soon made off at the unwonted disturbance of their quiet retreats.

We had time during our perambulations to direct our eyes to those who were intent on skating for its own sake. It appeared that our numbers comprised individuals in every stage of the art, from the tottering tyro upwards. Conspicuous for energy was a young clergyman, who, with his head forward, back bent, and long black coat-tails flying behind him, was dashing wildly about. But the culminating point was reached when he offered to assist our stout friend. She had never ventured on the ice before, and, indeed, seemed to overwhelm him so completely that it was not until she received very substantial assistance from the other side that he was able to murmur, in a weak voice, apologies for not having afforded more efficient help. He was one of several young men who, with the utmost gallantry and self-denial, made it their constant duty to assist one fair skater after another, and consequently were looked upon by the ladies as models of manly virtue.

*(To be continued.)*

